# Inhibition of Fungal Plant Pathogens by Synergistic Action of Chito-Oligosaccharides and Commercially Available Fungicides

**DOI:** 10.1371/journal.pone.0093192

**Published:** 2014-04-25

**Authors:** Md. Hafizur Rahman, Latifur Rahman Shovan, Linda Gordon Hjeljord, Berit Bjugan Aam, Vincent G. H. Eijsink, Morten Sørlie, Arne Tronsmo

**Affiliations:** Department of Chemistry, Biotechnology and Food Science, Norwegian University of Life Sciences (NMBU), Ås, Norway; Louisiana State University, United States of America

## Abstract

Chitosan is a linear heteropolymer consisting of β 1,4-linked *N*-acetyl-D-glucosamine (GlcNAc) and D-glucosamine (GlcN). We have compared the antifungal activity of chitosan with DP_n_ (average degree of polymerization) 206 and *F*_A_ (fraction of acetylation) 0.15 and of enzymatically produced chito-oligosaccharides (CHOS) of different DP_n_ alone and in combination with commercially available synthetic fungicides, against *Botrytis cinerea*, the causative agent of gray mold in numerous fruit and vegetable crops. CHOS with DP_n_ in the range of 15–40 had the greatest anti-fungal activity. The combination of CHOS and low dosages of synthetic fungicides showed synergistic effects on antifungal activity in both *in vitro* and *in vivo* assays. Our study shows that CHOS enhance the activity of commercially available fungicides. Thus, addition of CHOS, available as a nontoxic byproduct of the shellfish industry, may reduce the amounts of fungicides that are needed to control plant diseases.

## Introduction

*Botrytis cinerea* Pers.: Fr. (anamorph of *Botryotinia fuckeliana*) causes gray mold in over 200 plant species worldwide, which results in great damage to agricultural crops. For example, in Bangladesh, gray mold has caused near complete yield losses of chickpea [1] and in Norway the pathogen causes 30–60% yield reductions in strawberry production [2]. Other economically important plant pathogenic fungi include *Mucor piriformis* Fischer, causing postharvest rots on strawberries as well as on several other fruit crops [3]–[4], and *Alternaria brassicicola* (Schw.) Wiltshire, causing black spot on crucifers [5]. The control of these plant pathogens relies heavily on synthetic fungicides. Excessive use of synthetic fungicides has caused environmental pollution and development of fungicide resistance in plant pathogens [6]. Thus, there is a need to reduce the use of synthetic fungicides by increasing their efficacy or by finding alternatives.

Chitin, a linear biopolymer consisting of β 1,4-linked *N*-acetyl-D-glucosamine (GlcNAc) residues, is insoluble in water, aqueous acidic solutions and most organic solvents due to strong intra- and inter-chain hydrogen bonds [7]. The fraction of acetylation (*F*_A_) of chitin is usually above 0.90 [8], meaning that there are very few D-glucosamine (GlcN) units present. Chitosan, which is obtained by partial deacetylation of chitin, is a heteropolymer consisting of GlcNAc and GlcN residues. Chitosan with an *F*_A_ of around 0.65 or lower is soluble in aqueous acid solutions [7]–[9], Both chitin and chitosan can be hydrolyzed into chito-oligosaccharides (CHOS) by synthetic or enzymatic methods. CHOS are known to have several beneficial biological effects and may be used as fungicides, bactericides, bone-strengthener in osteoporosis, vector for gene delivery, hemostatic agent in wound-dressings, antimicrobial agents, and as inducer of plant defense responses against pathogens [10]–[12].

Hydrolysis of chitosan into CHOS can be done chemically or by glycosyl hydrolases (GH) classified as chitinases or chitosanases [13]. Chitinases are found in the GH families 18 and 19. Besides chitin, these enzymes also hydrolyze chitosans to varying extents, depending on the *F*_A_ [14]–[15]. Chitosanases are found in GH families 5, 7, 8, 46, 75 and 80 (see www.cazy.org for more details on the classification). Of these, the GH46, GH75 and GH80 families only contain chitosanases and the GH46 enzymes are probably the best studied. The key difference between chitinases and chitosanases is that only chitosanases can cleave GlcN-GlcN bonds and only chitinases can cleave GlcNAc- GlcNAc bonds. Apart from this clear difference the enzymes have varying and to some extent overlapping cleavage specificities that have been analyzed in several studies (the term “cleavage specificity” alludes to the specific sequences of GlcNAc and GlcN sugars that are being cleaved) [10].

Recently, we showed that CHOS fractions of DP_n_ 40 and DP_n_ 23 obtained from enzymatic hydrolysis of a chitosan (*F*_A_ = 0.15; DP_n_ = 206) by a family 46 chitosanase [16] significantly inhibited germination of isolates of *B. cinerea* and *M. piriformis* [17]. In the present study, we have investigated the antifungal effects that can be obtained by combining such CHOS with commercially available synthetic fungicides. To test anti-fungal effects, we have primarily studied inhibition of *B. cinerea in vitro* and *in vivo*, but effects on other fungal pathogens have also been addressed. Our results reveal remarkable synergistic effects of combining CHOS with synthetic fungicides, thus opening up new avenues towards the use of these oligosaccharides in environmentally benign plant protection strategies.

## Materials and Methods

### Fungal Cultures

*B. cinerea* (isolate BC 101), *A. brassicicola* (isolate A 328), and *M. piriformis* (isolate M119J) were obtained from the culture collection at the Norwegian University of Life Sciences (NMBU). For the *in vitro* and *in vivo* bioassays, conidia were collected from cultures grown on potato dextrose agar (PDA) (Difco Laboratories, Detroit, MI) under regular laboratory light for 2 weeks at 23±1°C. Concentrations of conidia in aqueous suspensions were determined by haemocytometer count at 400× magnification and adjusted to the required concentration with sterile water.

### Synthetic Fungicides

Five fungicides were tested: (1) Teldor® WG 50 (Bayer Crop Science Pty Ltd., Germany); active ingredient: 500 g kg^−1^ fenhexamid; chemical group: hydroxyanilide. (2) Switch® 62.5 WG (Syngenta Crop Protection Pty. Ltd., Switzerland); active ingredients: 375 g kg^−1^ cyprodinil and 250 g kg^−1^ fludioxonil; chemical groups: anilinopyramidine and phenylpyrrole respectively. (3) Amistar® (Syngenta Crop Protection Pty. Ltd.); active ingredient: 500 g kg^−1^ azoxystrobin; chemical group: strobilurin. (4) Signum® WG (BASF, Germany); active ingredients: 26.7% w/w boscalid and 6.7% w/w pyraclostrobin; chemical groups: pyridinecarboximide and methoxy-carbamate, respectively. (5) Delan® (BASF, Germany); active ingredient: 700 g kg^−1^ dithianon; chemical group: quinone.

### Enzymatic Production of CHOS

Chitosan (KitoNor, *F*_A_ 0.15, DP_n_ 206) was obtained from Norwegian Chitosan, Gardermoen, Norway. This chitosan was used for all experiments in this work. CHOS were produced by enzymatic hydrolysis of chitosan. Chitosanase ScCsn46A was produced as described by Heggset and coworkers [16]; briefly, the chitosanase, originally from *Streptomyces coelicolor* (UniProt accession code q9rj88), was purified from the culture supernatant of a recombinant *Escherichia coli* BL21Star (DE3) strain, following the published protocol, without removal of the (His)_6_-tag after purification. The enzyme was dialyzed against 20 mM Tris-HCl, pH 8.0, and stored at 4°C. Chitinase ChiA from *Serratia marcescens* was produced according to Brurberg and coworkers [18].

Chitosan (10 mg mL^−1^) in 0.04 M NaAc, 0.1 M NaCl, 1% HCl was incubated at 37°C and 225 rpm until the chitosan was dissolved (approximately 15 min). The pH was then adjusted to 5.5 with 0.5 M NaOH.

ScCsn46A [16] or ChiA [18] (0.5 μg mg^−1^ chitosan) were added to the chitosan solution and the mixture was incubated for various lengths of time at 37°C and 225 rpm. The enzymatic reaction was stopped by decreasing the pH to 2.5 with 0.5 M HCl, followed by immersing the tube in boiling water for at least 10 minutes to permanently inactivate the enzymes. CHOS samples were dialyzed against distilled water for 48 hours (water was changed every 12 hours) using a cellulose membrane (Float-A-Lyzer® MWCO 500 Da from Spectrum Labs, USA) to remove buffer salts from the sample. Dialyzed samples were sterile filtered through Filtropur S 0.2 μm sterile filters (Sarstedt, Germany), lyophilized and stored at 4°C [10]. ChiA was used to produce CHOS with predominantly GlcNAc on the reducing ends and ScCsn46A was used to produce CHOS with predominantly GlcN on the reducing ends (ChiA has an absolute preference for cleaving after GlcNAc [19]; ScCSn46 has a strong but not absolute preference for cleaving after a GlcN, and will essentially only cleave after GlcN under the conditions used here) [16]. It is important to note that the degree of degradation of chitosan cannot be monitored online (^1^H-NMR needs to be used; see below). This complicates reproducible production of CHOS batches with identical DP_n_ and explains why CHOS batches used in this study show slight variations in DP_n_.

### 1H-NMR Analysis °f CHOS

Lyophilized CHOS (10 mg) were dissolved in deuterium oxide (D_2_O) (0.5 mL) and the pH was adjusted to 4.2 with sodium deuteroxide (NaOD) prior to lyophilization. The lyophilized CHOS was redissolved in D_2_O and lyophilized again to secure that all the H_2_O had been removed. Finally the lyophilized CHOS were dissolved in D_2_O (700 μL) and ^1^H-NMR analysis was performed on a 300 MHz Varian Gemini instrument (Varian, USA) at 85°C. The DP_n_ was calculated by the equation (Dα+Dβ+D+Aα+Aβ+A)/(Dα+Dβ+Aα+Aβ), where Dα, Dβ, Aα and Aβ are the integrals of the reducing end signals of the α and β anomers of the deacetylated (D, GlcN) and acetylated (A, GlcNAc) units respectively, D is the integral of the signals from GlcN in internal positions and non-reducing end positions, and A is the integral of the signals from GlcNAc in internal and non-reducing end positions [14].

### Separation of CHOS by Size Exclusion Chromatography (SEC)

A CHOS sample (100 mg) generated by enzymatic hydrolysis of chitosan (DP_n_ 206) with ScCsn46A was applied to three Superdex™ 30 columns (XK columns from GE Healthcare) coupled in series with an overall dimension of 2.6×180 cm. The flow rate of the mobile phase (0.15 M NH_4_Ac, pH 4.5) was maintained at 0.8 ml min^−1^ [14]. A refractive index detector (Gilson model 133, UK) was used to monitor the relative amounts of the CHOS fractions.

### Effect of CHOS on Germination of B. cinerea, A. brassicicola and M. piriformis

Activity against *B. cinerea* was assessed using minimal salt medium (MSM) pH 5.2, with the following final concentrations: 2.5 mM NH_4_NO_3_; 0.28 mM CaCl_2_·2H_2_O; 0.16 mM MgSO_4_·7H_2_O; 0.002 mM MnSO_4_·4H_2_O; 0.002 mM ZnSO_4_·7H_2_O; 1 mM KH_2_PO_4_; 0.06 mM FeC_6_H_5_O_7_·5H_2_O and 55.5 mM glucose. Experiments were set up by adding 100 μL of CHOS or chitosan dissolved in 2×MSM to a 100 μL conidial suspension (2×10^4^ conidia mL^−1^ in water), in wells of a flat-bottom 96-well microtiter plate (Nunc™, Roskilde, Denmark). There were three replicate wells for each treatment. The microtiter plates were incubated at 23±1°C for 24 hours. Germination was visually estimated at 400×magnification using an invert microscope (Fluovert FU, Ernst Leitz Wetzlar GmbH, Wetzlar, Germany). The conidia were scored as germinated when the germ tube length was at least as long as the diameter of the conidium.

The germination inhibition percentage was calculated by the following equation:
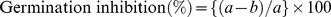



Where, a  =  number of germinated conidia in the control (conidia in MSM) b  =  germinated conidia in the treatment (conidia and chitosan/CHOS and/or fungicides in MSM).

The pH of the conidia suspension in the microtiter wells with and without CHOS was between 5.2 and 5.3 at the start of the experiment, and remained about the same 24 hours after inoculation.

Activity against *M. piriformis*, and *A. brassicicola* was tested in the same manner. Germinated *M. piriformis* M199J conidia showed abnormal swelling with amoeba-like structures and one or more protrusions. These conidia were counted as germinated if the length of at least one of the protrusions was at least as long as the diameter of the swollen conidia 12 hours after inoculation. Conidia of *A. brassicicola* were counted as germinated when the length of the germ tube was half of the conidia length.

### Synergism Between Fungicides and Chitosan or CHOS in Inhibiting *B. cinerea* and *M. piriformis in vitro*

Germination experiments were set up as described above, meaning that 100 μL of the to-be-tested samples were added to 100 μL of a conidia suspension in MSM. The samples were: a) control treatment (only MSM), b) chitosan or CHOS in MSM, c) chitosan or CHOS combined with synthetic fungicides (Teldor, Switch, Amistar or Signum) in MSM, and d) individual synthetic fungicides in MSM.

The interaction between synthetic fungicides and chitosan or CHOS was determined using Abbott's equation for synergy calculation [20]. The synergistic effect was calculated by determining the ratio between the observed efficacy E_obs_ (% inhibition) and the expected efficacy (E_exp_): E_exp_  =  a+b - (ab/100). Here a = % germination inhibition by synthetic fungicides (Teldor, Switch, Amistar or Signum) alone, b = % germination inhibition by chitosan or CHOS alone. An E_obs_/E_exp_ ratio equal to 1 indicates additivity, ratios >1 indicate synergy, and ratios <1 indicate an antagonistic interaction [20].

### *In Vivo* Bioassay: Inhibition of Infection of Strawberry Flowers and Chickpea Leaves By *B. cinerea*

Synergism between synthetic fungicides and chitosan or CHOS in inhibiting flower infection by *B. cinerea* was tested on newly opened strawberry (*Fragaria* × *ananassa*) flowers (cv. Corona). Strawberry plants were grown in a greenhouse with controlled temperature (18°C day; 12°C night), light (16 hours, light intensity: 150 μmols m^−2^ sec^−1^) and relative humidity (65%). Newly opened flowers were cut off with a 1½-2 cm stem and placed in empty pipette tip racks placed in plastic containers filled with 1–2 cm water. After mixing the conidia suspension (final concentration 1×10^6^ conidia mL^−1^) with each test solution, 10 μL drops of the mixtures were placed at the base of three petals on each flower using an automatic pipette (Finnpipette 4027, Thermo Labsystems, Finland). There were six replicates of three flowers (i.e., nine inoculation points per replicate) for each treatment. The sets of inoculated flowers were randomized and placed in containers covered with aluminium foil and incubated at 23±1°C. The relative humidity around the flowers was 90–95%, as measured using a thermo-hygrometer (Lambrecht, Germany). The experiments were repeated once. The infection incidence was determined visually: necrotic regions on the abaxial surface of the flowers (under the inoculation points) were registered daily for eight days and recorded as percent infected inoculation sites. The area under the disease progress curve (AUDPC) was calculated on the basis of the accumulated percent infection by the following equation:
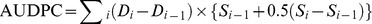
where *i*  =  number of assessment, *D_i_*  =  day of the i^th^ assessment and *S_i_*  =  percent infected inoculation points at the i^th^ assessment.

The protection index was calculated using the AUDPC values in the following formula [21]:
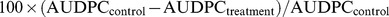
where *AUDPC*_control_ is derived from infection in flowers inoculated with *B. cinerea* conidia alone and *AUDPC*_treatment_ is derived from infection in flowers treated with synthetic fungicides and/or CHOS premixed with *B. cinerea* conidia.

Similar tests were performed using detached chickpea (*Cicer arientinum* L.) leaves. Chickpea were grown in the green house at 22±3°C under twelve hours light. Three compound chickpea leaves were used for each treatment and each chickpea leaf had one inoculation point on six of its leaflets. There were three replicates of each treatment. The chickpea leaves were inoculated with 10 μL drops of a 2×10^6^ mL^−1^ suspension of *B. cinerea* conidia in water, supplemented with sterile water (control) or solutions of the to-be-tested compounds in sterile water. The infection was recorded when a brown (necrotic) spot appeared under the inoculation point, and the cumulative disease development was recorded daily up to eight days after inoculation.

Sporulation of *B. cinerea* on the chickpea leaves was recorded at the end of the experiment. To do so all leaves from each treatment were soaked in sterile water (10 mL) for 20 min at 25°C and vortexed several times. Subsequently, the conidia concentration in the water was determined by counting in a hemacytometer.

### Field trial: Inhibition of Infection of Apple Fruits by *Venturia ineuqualis*

Apple trees (*Malus domestica* Broch) of the cultivar Aakerø in the apple orchard at the Norwegian University of Life Sciences, Ås, Norway were used. The experiment was conducted in 2013 and there were three replicates of each treatment and three trees in each replicate. The trees were sprayed to runoff once in the flowering period (28^Th^ of May) and three times in the fruiting season (24^th^ of June, 7^th^ of July and 17^th^ of August). At harvest (3^rd^ of September) the fraction of apples with infection of apple scab (*Venturia inaequalis*) was recorded.

### Data Analysis

In the microtiter plate assay, the percentages of germination inhibition of pathogens by chitosan and CHOS were transformed by arcsine transformation and tested by one way ANOVA (only non transformed data are presented). In the strawberry flower assay, the AUDPC was calculated based on cumulative daily infection from one to eight days, and tested by one way ANOVA. When appropriate, means were separated by Tukey's Honestly Significant Difference method. All statistical analysis was done using Microsoft Office Excel 2007 and Minitab 16 (MINITAB, USA).

## Results

### Enzymatic Production of CHOS

CHOS were produced by degrading chitosan (DP_n_ of 206 and *F*_A_ of 0.15) with either ChiA or ScCsn46A, as described above. By varying the incubation time CHOS fractions with DP_n_ values between 96 and 9 could be obtained. Note that the determination of DP_n_ needs to be done (by NMR) after the enzymatic reaction has been concluded, explaining why it is difficult to produce CHOS fractions with exactly the same DP_n_. Since there are indications in the literature that the biological effects of CHOS depend not only on DP and *F*_A_, but also on the pattern of acetylation [10] we initially tested the effect of the only controllable aspect of this pattern, namely the sugar at the reducing end of the CHOS. Hydrolysis by ChiA yields GlcNAc at the reducing end, whereas ScCsn46A almost exclusively yields GlcN.

### Effect of the Reducing End Sugar on the Ability of CHOS To Inhibit Germination of *B. cinerea*

**Figure 1:**
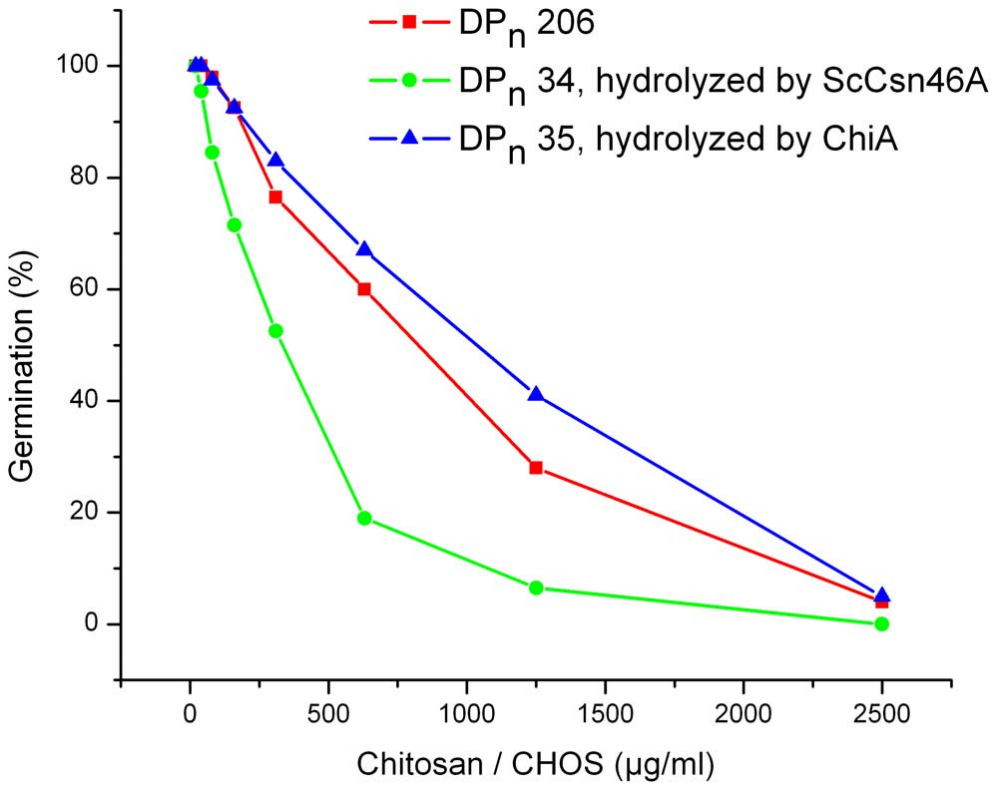
Effect of the reducing end sugars (GlcN vs GlcNAc) on the ability of CHOS to inhibit germination of *B. cinerea* (measured 24 hours after inoculation). Squares: chitosan, DP_n_ 206, 85 % D at the reducing end; circles: CHOS generated by ScCsn46A, DP_n_ 33.5, >90% GlcN at the reducing end; triangles: CHOS generated by ChiA, DP_n_ 34.6, about 35% GlcNAc at the reducing end. Data points represent the mean of three replicate wells.

To test the effect of the reducing end sugars (GlcN vs GlcNAc) on the antifungal activity of CHOS, we tested the efficacy of chitosan (DP_n_ 206, 85% GlcN at the reducing ends), CHOS DP_n_ 33.5 prepared with ScCsn46A (>90% GlcN at the reducing ends), and CHOS DP_n_ 34.6 prepared with ChiA (about 35% GlcNAc at the reducing end). Figure 1 shows that CHOS produced with ScCsn46A were more effective than CHOS produced with ChiA. Based on these observations all further studies were done with CHOS obtained from degradation of chitosan (DP_n_ 206; *F*_A_ 0.15) with ScCsn46A.

### The Effect of the Degree of Polymerization on Inhibition of Germination of Fungal Conidia

**Figure 2:**
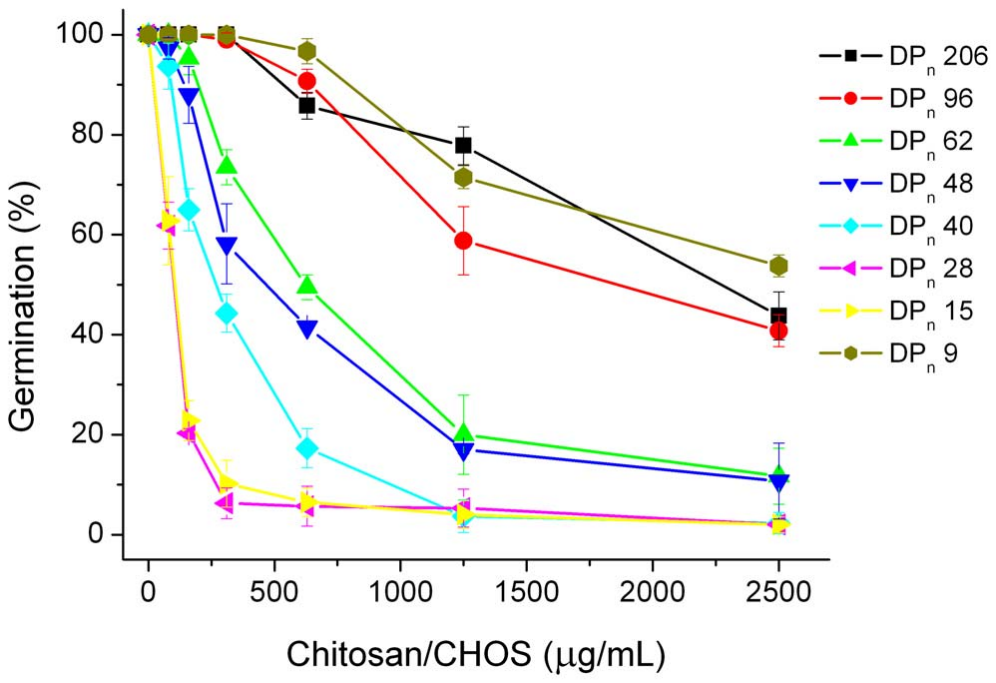
Effect of chitosan (DP_n_ 206) or CHOS obtained by hydrolysis of chitosan with ScCsn46A on germination of *Botrytis cinerea* (measured 24 hours after inoculation). The data points are the mean of three experiments with standard deviation.

Studies of the inhibitory effect of chitosan/CHOS with different DP_n_ (206 – 9) on *B. cinerea* germination showed that the most active fractions of CHOS had DP_n_ values around 28, but that also other CHOS samples with DP_n_ values in the range of 15 to 40 had good antifungal activities. All tested CHOS fractions (except DP_n_ 9) were more inhibitory than the chitosan (Fig. 2).

**Figure 3:**
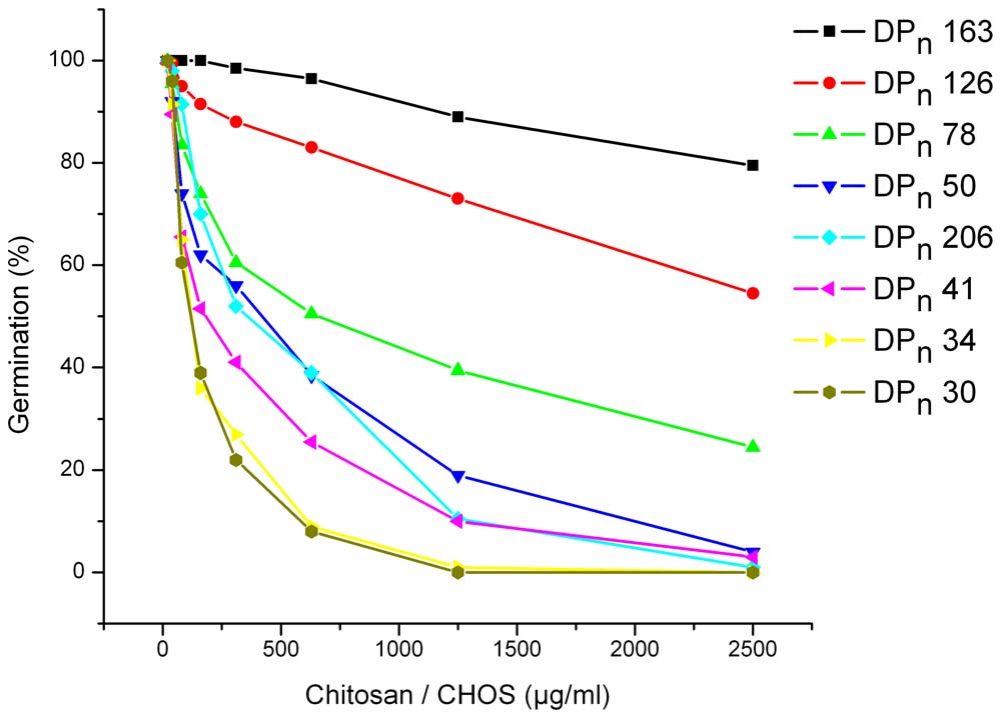
Dose-response relationships for the inhibitory effect of chitosan DP_n_ 206 and various CHOS fractions on germination of *Botrytis cinerea* (measured 24 hours after inoculation). CHOS DP_n_ 34 was produced by hydrolysis of chitosan (DP_n_ 206) with ScCsn46A. CHOS DP_n_ 34 was separated by size exclusion chromatography to fraction with DP_n_ 30, 41, 50, 78, 126 and 163.

To investigate the antifungal effect of CHOS with a narrower range of chain lengths than could be obtained by hydrolyzing with ScCsn46A for various lengths of time, a CHOS mixture obtained by hydrolyzing chitosan (DP_n_ 206) with ScCsn46A to DP_n_ 34 was sub fractionated using size exclusion chromatography. The DP_n_ values of the resulting CHOS fractions were determined using NMR. Figure 3 shows that the fractions with DP_n_ in the range of 78 – 163 were less inhibitory to *B. cinerea* than the starting material chitosan, whereas the most inhibitory CHOS fractions were those with DP_n_ 30 and 34.

**Figure 4:**
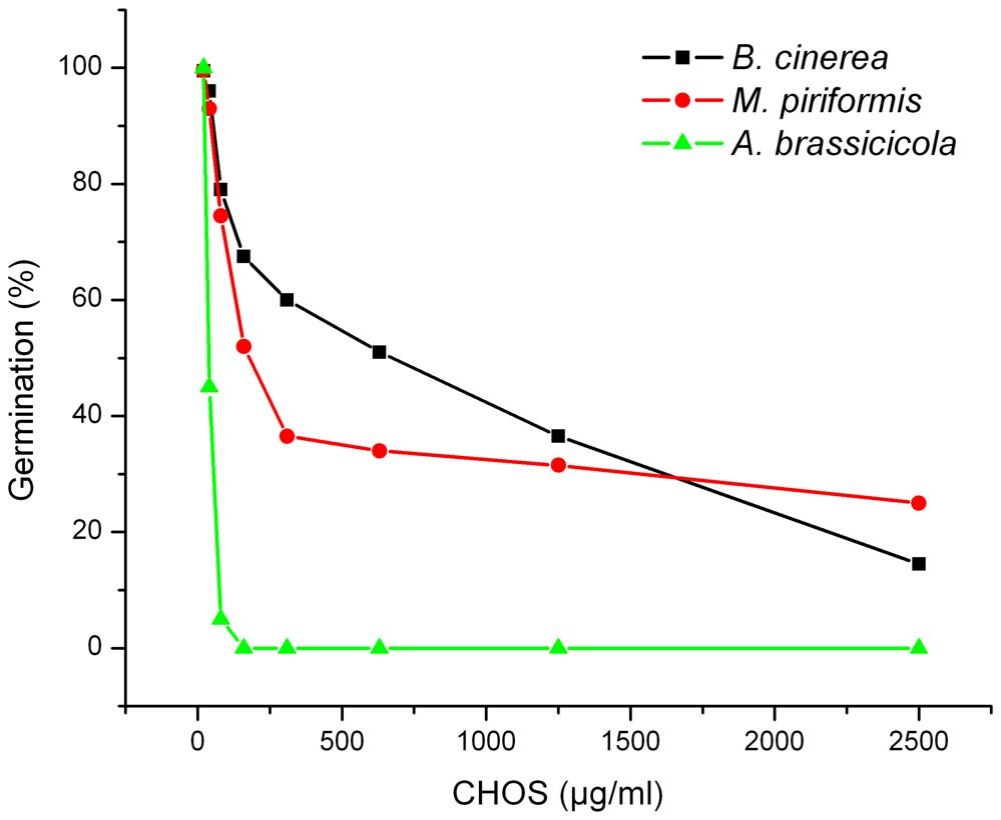
Dose-response relationships for the inhibitory effect of CHOS (DP_n_ 37) on germination of *Botrytis cinerea*, *Alternaria brassicicola* and *Mucor piriformis* (measured 24 hours after inoculation).

In another set of experiments, the effects of a CHOS fraction with DP_n_ 37 on germination of plant pathogenic fungi belonging to three different genera were tested. The three fungi showed quite different dose-response relationships (Fig. 4). While *B. cinerea* and *M. piriformis* showed decreasing germination over a broad concentration range of CHOS (20–2500 μg mL^−1^), *A. brassicicola* was completely inhibited by 80 μg mL^−1^ CHOS. 50% germination inhibition of *A. brassicicola* was obtained at 40 μg mL^−1^, whereas CHOS concentrations of 630 μg mL^−1^ and 160 μg mL^−1^ were needed to obtain 50% inhibition of *B. cinerea* and *M. piriformis*, respectively.

### *In Vitro* Testing of the Effects of Combining Synthetic Fungicides with Chitosan or CHOS

Table 1 shows germination-inhibition data for *B. cinerea* treated with chitosan or a combination of chitosan and one of four synthetic fungicides, Teldor, Switch, Amistar or Signum. The data show minor effects when adding chitosan alone (10% inhibition, at 80 μg mL^−1^), and reveal small synergistic effects for most of the combinations (Table 1). In the case of Teldor, however, quite strong synergistic effects were observed. For example, while application of 60 μg mL^−1^ Teldor alone gave only 1.3% inhibition, co-administration with 80 μg mL^−1^chitosan yielded as much as 64% inhibition.

**Table 1:** Inhibition of germination of *Botrytis cinerea* by chitosan (DP_n_ 206) and synthetic fungicides, alone and in combination.

Treatment (μg mL^−1^)	Germination inhibition (% ± SD)^a^	E_obs_/E_exp_ b
Chitosan 80	10.4±2.5	-
Teldor 60	1.3±0.6	-
Teldor 15	1.3±0.6	-
Chitosan 80 + Teldor 60	64.2±8.9	4.8
Chitosan 80 + Teldor 15	35.6±5.2	3.0
		-
Switch 25	74.8±6.0	-
Switch 5	35.3±4.2	-
Chitosan 80 + Switch 25	91.6±7.8	1.2
Chitosan 80 + Switch 5	56.2±7.9	1.3
		-
Amistar 100	20.0±4.5	-
Amistar 10	3.6±3.6	-
Chitosan 80 + Amistar 100	31.7±1.7	1.0
Chitosan 80 + Amistar 10	14.8±3.8	1.1
		-
Signum 10	17.7±1.5	-
Signum 2	3.8±3.1	-
Chitosan 80 + Signum 10	24.7±4.2	1.1
Chitosan 80 + Signum 2	17.8±5.5	1.3

Germination was recorded 24 hours after inoculation.

a All data are the mean of three experiments ± standard deviation

b An E_obs_/E_exp_ ratio of 1 indicates additivity; ratios >1 indicate synergy.

Strikingly, similar experiments on inhibition of germination of *B. cinerea* with CHOS (DP_n_ 23) showed large synergistic effects for almost all combinations of CHOS (DP_n_ 23) and the synthetic fungicides (Table 2). While CHOS alone (5 μg mL^−1^) and the synthetic fungicides alone, each applied at low concentrations, generally only slightly inhibited germination, in several cases more than 90% inhibition could be obtained by combining the two types of anti-fungal compounds. For example Amistar (10 μg mL^−1^) and Signum (10 μg mL^−1^) applied alone gave only 1.6% germination inhibition; upon addition of CHOS (5 μg mL^−1^; yielding 4.8% inhibition when applied alone), germination inhibition increased to 96% and 93%, respectively (Table 2).

**Table 2:** Effect of chitooligosaccharides (CHOS DP_n_ 23) and synthetic fungicides on germination inhibition of *Botrytis cinerea*.

Treatment (μg mL^−1^)	Germination inhibition (% ± SD)^a^	E_obs_/E_exp_ b
CHOS 5	4.8±3.7	-
Teldor 150	4.4±4.2	-
Teldor 15	0.6±0.9	-
CHOS 5 + Teldor 150	21.0±5.1	2.3
CHOS 5 + Teldor 15	21.7±5.8	4.0
		
Switch 25	81.7±4.9	-
Switch 5	18.3±11.7	-
CHOS 5 + Switch 25	94.2±5.7	1.1
CHOS 5 + Switch 5	96.2±2.7	4.3
		
Amistar 100	4.6±1.7	-
Amistar 10	1.6±0.7	-
CHOS 5 + Amistar 100	95.6±3.4	10.4
CHOS 5 + Amistar 10	96.4±3.6	15.3
		
Signum 10	1.6±0.8	-
Signum 2	1.7±1.7	-
CHOS 5 + Signum 10	93.2±7.1	16.5
CHOS 5 + Signum 2	89.0±7.0	15.8

Germination was recorded 24 hours after inoculation.

a All data are the mean of three experiments ± standard deviation

b An E_obs_/E_exp_ ratio of 1 indicates additivity; ratios >1 indicate synergy.

### *In Vivo* Testing of the Effects of Combining Synthetic Fungicides with Chitosan or CHOS

In the strawberry flower assay, chitosan (400 μg mL^−1^) gave approximately the same level of protection against *B. cinerea* as the synthetic fungicides applied at 1% of the recommended dose (Table 3). Clear synergistic effects were not observed. Interestingly though in one case (Amistar), the combination of the synthetic fungicide at 1% of the recommended dose and chitosan (400 μg mL^−1^) yielded a level of protection that was similar to the protection level achieved by the recommended dose of fungicide (Table 3).

**Table 3:** Inhibition of disease development in strawberry flowers inoculated with a mixture of *Botrytis cinerea* conidia and chitosan (DP_n_ 206) and/or synthetic fungicides.

Treatment (μg mL^−1^)	AUDPC (± SD)^a^	Protection index (% ± SD)^a^^,^ b
Control^c^	5.0±0.2	-
Chitosan 400	3.8±0.2	24±3
Teldor 1500^d^	1.5±0.3	70±6
Teldor 15	3.8±0.5	23±7
Chitosan 400 + Teldor 15	2.4±0.2	53±11
		
Switch 500^d^	± 0.2	80±5
Switch 5	3.2±1.0	36±10
Chitosan 400 + Switch 5	2.1±0.3	58±4
		
Amistar 1000^d^	2.0±0.3	60±5
Amistar 10	3.5±0.1	31±2
Chitosan 400 + Amistar 10	2.0±0.3	60±4
		
Signum 1000^d^	1.3±0.3	74±5
Signum 10	3.7±0.2	26±7
Chitosan 400 + Signum 10	2.5±0.1	50±4

Disease development was scored as development of visual necrotic regions under the inoculation point up to eight days after inoculation and is quantified as the area under the disease progress curve (AUDPC). The protection index was calculated on the basis of the AUDPC values.

a All data are the mean of two experiments ± standard deviation with 6×3 flowers in each treatment.

b The AUDPC was used to calculate the protection index.

c Conidia in sterile water.

d Recommended dose.

Like chitosan (DP_n_ 206, 400 μg mL^−1^), CHOS (DP_n_ 23, at the low concentration of 10 μg mL^−1^) hardly inhibited flower infection by *B. cinerea*, but combinations of CHOS with the synthetic fungicides revealed large synergistic effects and showed that effective inhibition of infection could be achieved with low concentrations of both CHOS and synthetic fungicides (Table 4). When co-administrated with 10 μg mL^−1^ CHOS, the protection levels achieved with the synthetic fungicides at 1% of the recommended concentration were 80%, 92%, 80% and 85% for Teldor, Switch, Amistar and Signum, respectively.

**Table 4:** Inhibition of disease development in strawberry flowers inoculated with a mixture of *Botrytis cinerea* conidia and chitooligosaccharides (CHOS DP_n_ 23) and/or synthetic fungicides.

Treatment (μg mL^−1^)^a^	AUDPC (± SD)^b^	Protection index (% ± SD)^b^^,^ c	E_obs_/E_exp_
Control^d^	4.7±0.2	-	-
CHOS 10	4.4±0.2	5±3	-
Teldor 150	2.8±0.5	39±11	-
Teldor 15	4.4±0.1	5±1	-
CHOS 10 + Teldor 150	0.6±0.2	87±5	2
CHOS 10 + Teldor 15	0.9±0.4	80±8	8
			-
Switch 25	4.3±0.1	9±3	-
Switch 5	4.5±0.2	3±1	-
CHOS 10 + Switch 25	0.6±0.4	87±4	6
CHOS 10 + Switch 5	0.4±0.4	92±8	12
			-
Amistar 100	4.5±0.2	3±1	-
Amistar 10	4.6±0.2	1±1	-
CHOS 10 + Amistar 100	0.9±0.3	79±8	10
CHOS 10 + Amistar 10	0.9±0.4	80±10	13
			-
Signum 10	4.4±0.1	4±2	-
Signum 2	4.6±0.1	2±1	-
CHOS 10 + Signum 10	0.7±0.3	85±7	10
CHOS 10 + Signum 2	0.6±0.4	86±8	12

Disease development and protection index were scored as in Table 3. The synergistic effect was calculated by determining the ratio between the observed efficacy E_obs_ (% inhibition) and the expected efficacy (E_exp_) (see materials and methods). An E_obs_/E_exp_ value of 1 indicates additivity, while E_obs_/E_exp_>1 indicates synergy.

a The recommended doses for the synthetic fungicides are 1500, 500, 1000 and 1000 μg mL^−1^ for Teldor, Switch, Amistar and Signum, respectively.

b All data are the mean of two experiments ± standard deviation, with 6×3 flowers in each treatment.

c The AUDPC was used to calculate the protection index.

d Conidia in sterile water.

**Figure 5:**
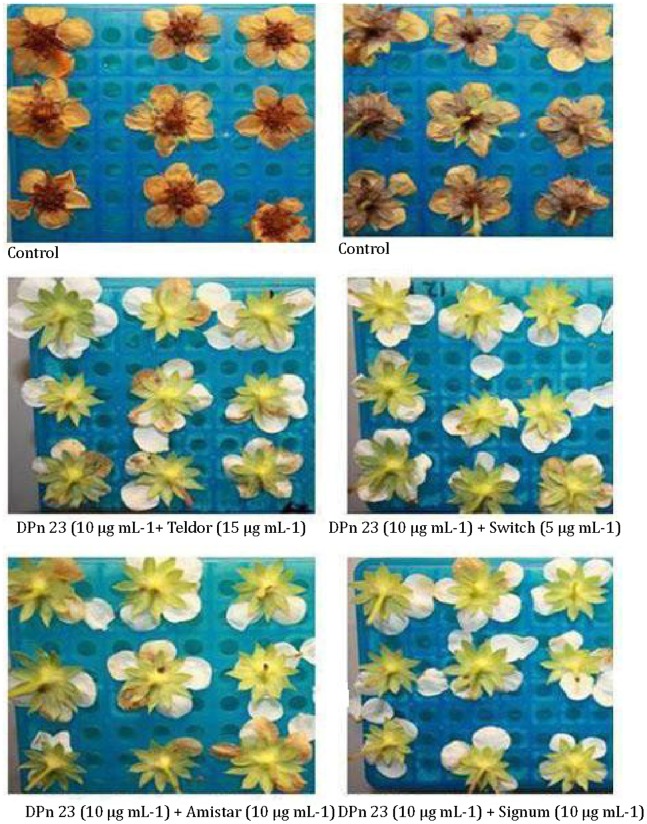
Combined anti-fungal effects of CHOS and synthetic fungicides. The pictures illustrate the inhibitory effects of combinations of a synthetic fungicide (Teldor, Switch, Amistar or Signum, at 15, 5, 10 and 10 μg mL^−1^, respectively) and CHOS (DP_n_ 23, 10 μg mL^−1^) on disease caused by *Botrytis cinerea* applied to detached strawberry flowers, six days after inoculation. The flowers were considered 100% infected when all three inoculation points displayed necrotic signs. All treatments included 18 flowers, but only nine flowers are shown. Control flowers were inoculated with conidia in sterile water.

In the control treatment (no anti-fungal compounds added) 100% of the strawberry flowers showed signs of infection 3 – 4 days after inoculation and a similar result was obtained when CHOS (DP_n_ 23, 10 μg mL^−1^), Teldor (15 μg mL^−1^), Switch (5 μg mL^−1^), Amistar (10 μg mL^−1^) or Signum (10 μg mL^−1^) were applied alone. However, when the inoculated flowers were treated with combinations of CHOS (DP_n_ 23) and synthetic fungicides (at the mentioned concentrations) no visible infection occurred before six days after inoculation (Fig. 5).

In a chickpea leaf bioassay, chitosan, CHOS (DP_n_ 30) and Switch were used alone and in combination against *B. cinerea* (Table 5). The combinations of chitosan (320 μg mL^−1^) or CHOS DP_n_ 30 (320 μg mL^−1^) and Switch (1% of the recommended dose) showed synergism, albeit less strongly than in the strawberry flower assay. The combination of CHOS (320 μg mL^−1^) and Switch (10 μg mL^−1^) was almost as protective (96%) as the recommended dose of Switch (500 μg mL^−1^; 98% protection). CHOS consistently showed better effects than chitosan.

**Table 5:** Effect of combinations of chitosan (DP_n_ 206) or chito-oligosaccharides (CHOS DP_n_30) and Switch on *Botrytis cinerea* infection of detached chickpea leaves.

Treatment (μg mL^−1^)	AUDPC (± SD)^a^	Protection index (% ± SD)^a^^,^ b	E_obs_/E_exp_ c
Control^d^	6.5	-	-
Chitosan 2500	4.4±0.4	33±7	-
Chitosan 320	6.1±0.2	5±2	-
CHOS 2500	2.8±0.4	58±7	-
CHOS 320	5.5±0.1	15±2	-
Switch 500^e^	0.1±0.1	98±1	-
Switch 10	1.3±0.5	80±7	-
Switch 5	3.5±0.4	46±6	-
Chitosan 320 + Switch 10	1.4±0.1	79±2	1
Chitosan 320 + Switch 5	1.3±0.2	80±4	2
CHOS 320 + Switch 10	0.3±0.1	96±1	1
CHOS 320 + Switch 5	0.7±0.3	90±5	2

Disease development was scored daily up to eight days after inoculation.

a All data are the mean of three replicates (each replicate contained three compound leaves with 6 inoculated leaflets) ± standard deviation.

b The AUDPC was used to calculate the protection index.

c E_obs_/E_exp_ 1 indicates additivity; E_obs_/E_exp_>1 indicates synergy.

d Conidia in sterile water.

e Recommended dose.

**Figure 6:**
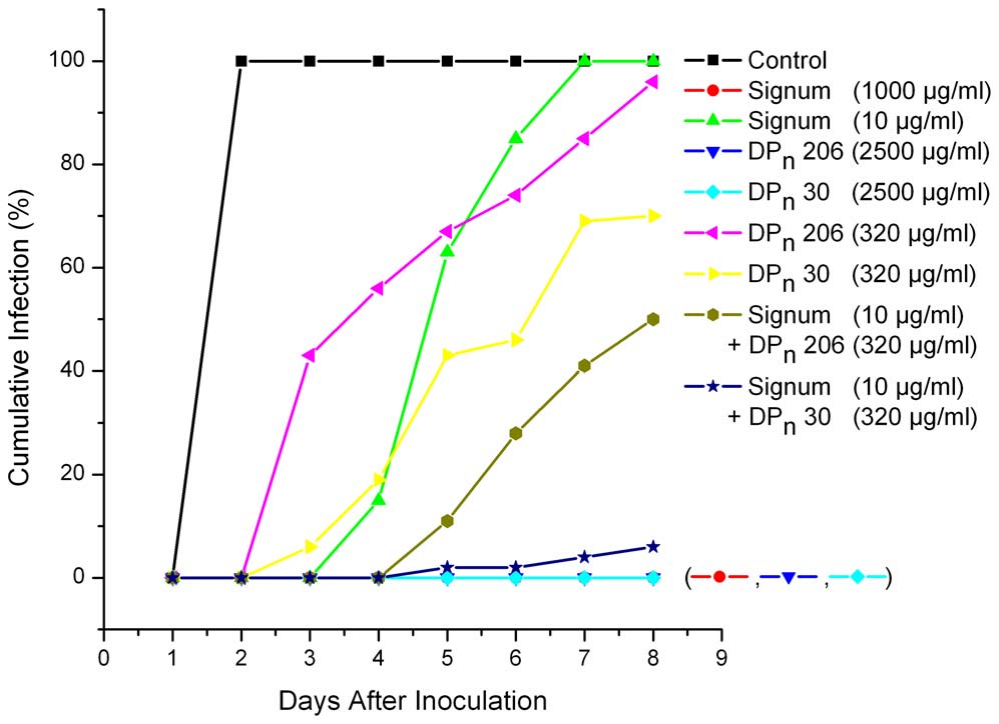
Effect of combinations of chitosan (DP_n_ 206) or CHOS (DP_n_ 30) and Signum on cumulative *Botrytis cinerea* infection of detached chickpea leaves.

Similar studies with Signum (Fig. 6) showed no synergistic effects, but the effects of chitosan (320 μg mL^−1^) or CHOS DP_n_ 30 (320 μg mL^−1^) and Signum (5 or 10 μg mL^−1^) were additive, meaning that also in this case chitosan or CHOS may be used to reduce usage of the synthetic fungicide. For example, the combination of 320 μg mL^−1^ CHOS (DP_n_ 30) and 10 μg mL^−1^ Signum (1% of recommended dose) yielded 98% inhibition. Again, CHOS consistently showed better effects than chitosan.

Sporulation of the plant pathogenic fungus on infected plant parts is an important source of secondary infections. Therefore, experiments were performed to assess the effects of the combination of chitosan or CHOS (DP_n_ 30) with Signum on the sporulation of *B. cinerea* on infected chickpea leaves. As shown in Table 6, combinations of chitosan or CHOS (DP_n_ 30) and Signum reduced sporulation of *B. cinerea* more than each component alone. Of the tested conditions, the combination of CHOS (320 μg mL^−1^) and Signum (10 μg mL^−1^) was the most effective.

**Table 6:** Effect of combinations of chitosan (DP_n_ 206) or chito-oligosaccharides (CHOS; DP_n_ 30) and Signum on sporulation of *Botrytis cinerea* on infected chickpea leaves.

Treatment (μg mL^−1^)	Conidia (10^3^ mL^−1^)^b^
Control^a^	290±92
Signum 10	52±12
Chitosan 320	110±32
CHOS 320	57±17
Chitosan 320 + Signum 10	11±3
CHOS 320 + Signum 10	0.4±0.1

Spores were counted eight days after inoculation.

a Conidia in sterile water.

b The data are the mean of two experiments ± standard deviation. Each experiment had three replicates for each treatment and each replicate had three leaves with 6 inoculated leaflets.

### Field trial: Inhibition of Infection of Apple Fruits by *Venturia ineuqualis*

In a field trial we studied the effect of 0.1% (w/v) CHOS DP_n_ 35 combined with Delan at recommended concentration (0.8% w/v)) or at 1/10 of the recommended concentration (0.08% w/v) on development of scab in apples, which is due to infection by *Venturia inaequalis*.

The results in Table 7 show that the combination of CHOS and 1/10 of the recommended concentration of Delan was more effective in preventing scab development than the recommended concentration of Delan.

**Table 7:** Effect of the combination of chito-oligosaccharides (CHOS; DP_n_ 35) and Delan on infection of apple by *Venturia inaequalis* in the field.

Treatment	% apples with apple scab
Untreated control	31.2±9.7^a^
Delan 0.8 g/L (800 μg ml^−1^)^b^	20.9±9.5
Delan 0.08 g/L (80 μg ml^−1^)	27.5±12.0
CHOS DP_n_ 30, 1.0 g/L (1000 μg ml^−1^)	25.9±13.3
Delan 80 μg ml^−1^ + Chitosan DP_n_ 30, 1000 μg ml^−1^	16.7±5.2

a Standard deviation. The data are derived from one experiment (one season) with three replicates per treatment and three trees in each replicate.

b Recommended dose.

## Discussion

It is well known from several studies that chitosan and CHOS have anti-microbial properties, and it is also known that the degree of acetylation of chitosan is an important factor affecting antifungal activity [22]–[23]. It has been proposed that the positive charge of the free amino groups of the glucosamine moieties in chitosan modulates interactions with the negatively charged cell surface, which under certain conditions may result in membrane destabilization and pore formation [22]–[23]. In the present study, we have focused on the effects of chain length, the particular role of the sugar moiety at the reducing end, and, first of all, on synergistic effects between chitosan or CHOS and synthetic fungicides.

To our knowledge there are no previous reports showing what is presented above, namely that the presence of GlcN at the reducing ends of CHOS is beneficial for antifungal activity. Interestingly, a common method to produce CHOS from chitin or chitosan is to treat the polymers with concentrated HCl in an acid catalyzed hydrolysis [24]. Due to the intrinsic chemistry of this reaction, hydrolysis after an acetylated sugar is favored 115 times more than hydrolysis after a deacetylated sugar [24]. Taking into account the beneficial effect of a deacetylated sugar at the reducing end on anti-fungal activity, chemical hydrolysis of chitosan could give less effective CHOS than hydrolysis using an enzyme such as ScCsn46A.

It has been suggested that CHOS are more inhibitory than polymeric chitosan due to better solubility in water [25]–[26]. The present results shows that the degree of polymerization (DP) is an important factor on the antifungal activity. Since all the chitosans and CHOS used in this study (with low F_A_ and at slightly acidic pH) are almost equally soluble at pH 5.3, it is unlikely that the antifungal activity of the chitosan and CHOS tested can be explained by a slight difference in water solubility.

Our *in vitro* assay showed that CHOS obtained using ScCsn46A were more inhibitory toward *B. cinerea* than the native chitosan (DP_n_ 206). CHOS in the DP_n_ range 15–40 were the most effective. The dosages were calculated by weight, rather than by moles, and thus the molar concentration of the smaller CHOS was higher than that of the longer CHOS. However, if one converts the data shown in Fig. 2 to molar dosages, the data still show a clear optimal DP_n_ in the region 15 – 40 (Note that the inhibitory effect becomes strongly reduced at DP_n_<15). Interestingly, a previous study on the effect of CHOS on *Candida krusei* (the tested range was 5 to 27 kDa) [27] showed that antifungal activity was at is maximum for a 6 kDa CHOS fraction (DP_n_ around 40), whereas longer CHOS were less effective. The present results are in accordance with this observation.

It is of interest to note that the longer CHOS obtained after fractionating a CHOS sample with DP_n_ 34 by size exclusion chromatography were less inhibitory than the original chitosan (DP_n_ 206) (Figure 3). This indicates that the shorter CHOS molecules likely to be present in the chitosan DP_n_ 206, but not in the chromatographically purified DP_n_ 78, 126 and 163 fractions, are important for the antifungal activity.

The most important results of the present study is the demonstration of good effects of combining CHOS or chitosan with synthetic fungicides, which was observed in vitro and in vivo laboratory studies as well as in a field trial. In all cases, additivity was observed and in several cases the combinations were strongly synergistic in both *in vitro* and *in vivo* assays. The effects varied between the various fungi and plants tested, but the overall picture is that synergistic effects are common and that CHOS of the right DP_n_ tend to work better than chitosan, sometimes much better. The largest synergistic effects were observed with *B. cinerea*, in both the germination assay and the strawberry flower assay (Tables 2 & 4). For example, low concentrations of CHOS (DP_n_ 23) or Signum, which had almost no effect on *B. cinerea* germination when applied separately, achieved almost 90% reduction of germination when applied together.

The mechanisms for the synergism in inhibition of fungal growth are not known. Most likely, the synergism is due to the compounds' different modes of action. Teldor inhibits sterol biosynthesis, Switch inhibits protein synthesis and signal transduction, while Amistar and Signum inhibit respiration [28]. The mode of action of chitosan is not clearly understood [29]–[32] but previous studies suggest that electrostatic interactions between positively charged chitosan and the negatively charged cell surface may destabilize the cell wall and/or cell membrane, which ultimately increases the cell permeability and induces cell leakage [33]–[35]. The synergy could conceivably be the result of a general increase in stress when different cellular processes are attacked simultaneously. More specifically, increased cell wall permeability may have enabled Teldor (fenhexamid) to reach the conidial membrane earlier and thereby stop the germination at an earlier stage than if Teldor was applied alone. Increased cell membrane permeability [35] may enable Amistar and Signum to inhibit respiration or Switch to inhibit protein synthesis more easily than if the fungicides are applied alone. The reasons for the stronger synergism between CHOS and fungicides compared to chitosan and fungicides are not known, but this observation correlates with the observed clear optimum in chain length that was observed when applying CHOS alone (Fig. 2).

An issue not addressed in the present study but of major interest for future work concerns possible interactions between the CHOS and CHOS-binding proteins in the plant or the pathogenic fungus, in particular proteins containing LysM domains [36]. CHOS can stimulate plant immune responses by binding to specific receptor proteins, and such stimulation could contribute to the observed overall protective effects of CHOS and CHOS-fungicide mixtures. On the other hand plant pathogenic fungi may combat this response by secreting proteins that sequester CHOS [37], which could reduce protective effects. It is thus conceivable that variation in the protective effects described above to some extent is due to variation in the interactions between the CHOS and CHOS-binding proteins in plant or fungus. Notably, the *in vitro* data show strong anti-fungal effects of CHOS-fungicide mixtures, which suggests that direct inhibition of fungal growth is a dominant contributor to the protective effects seen in the *in vivo* experiments.

In conclusion, our studies suggest that the use of CHOS of DP_n_ 15–40, with a deacetylated reducing end may reduce the need for synthetic fungicides by at least an order of magnitude. Thus, combinations of CHOS and synthetic fungicides should be considered for use in Integrated Pest Management (IPM) programs, where application of even small amounts of CHOS could reduce the need for synthetic fungicides considerably.
